# The politics of scaling

**DOI:** 10.1177/03063127211048945

**Published:** 2021-10-08

**Authors:** Sebastian Pfotenhauer, Brice Laurent, Kyriaki Papageorgiou, and Jack Stilgoe

**Affiliations:** 1 Technical University of Munich, München, Germany; 2 MINES ParisTech, PSL Research University, CSI – Centre de Sociologie de l’Innovation, i3 UMR CNRS, Paris, France; 3 ESADE Business School, Sant Cugat, Spain; 4 University College London, London, UK

**Keywords:** scale, innovation, platform technologies, living labs, randomized controlled trials

## Abstract

A fixation on ‘scaling up’ has captured current innovation discourses and, with it, political and economic life at large. Perhaps most visible in the rise of platform technologies, big data and concerns about a new era of monopolies, scalability thinking has also permeated public policy in the search for solutions to ‘grand societal challenges’, ‘mission-oriented innovation’ or transformations through experimental ‘living labs’. In this paper, we explore this scalability zeitgeist as a key ordering logic of current initiatives in innovation and public policy. We are interested in how the explicit preoccupation with scalability reconfigures political and economic power by invading problem diagnoses and normative understandings of how society and social change function. The paper explores three empirical sites – platform technologies, living labs and experimental development economics – to analyze how scalability thinking is rationalized and operationalized. We suggest that social analysis of science and technology needs to come to terms with the ‘politics of scaling’ as a powerful corollary of the ‘politics of technology’, lest we accept the permanent absence from key sites where decisions about the future are made. We focus in on three constitutive elements of the politics of scaling: solutionism, experimentalism and future-oriented valuation. Our analysis seeks to expand our vocabulary for understanding and questioning current modes of innovation that increasingly value scaling as an end in itself, and to open up new spaces for alternative trajectories of social transformation.

I meant no harm I most truly did not,

but I had to grow bigger so bigger I got.

I biggered my factory, I biggered my roads,

I biggered the wagons, I biggered the loads,

of the Thneeds I shipped out I was shipping them forth

from the South, to the East, to the West, to the North.

I went right on biggering … selling more thneeds.

And I biggered my money which everyone needs.

From *The Lorax*, by Dr. Seuss

## A scalability zeitgeist

In 2014, tech billionaire and investor Peter Thiel’s published his start-up manifesto ‘Zero to One’, in which he unabashedly put the appetite for scaling and monopoly at the heart of technological entrepreneurship:

Every single company in a good venture portfolio must have the potential to succeed at vast scale. … Everyone needs to know exactly one thing that even venture capitalists struggle to understand: we don’t live in a normal world; we live under a power law. … The power law becomes visible when you follow the money: in venture capital, where investors try to profit from exponential growth in early-stage companies, a few companies attain exponentially greater value than all others. … The most successful companies make the core progression – to first dominate a specific niche and then scale to adjacent markets – a part of their founding narrative. (Thiel, 2014)

**Figure 1.**
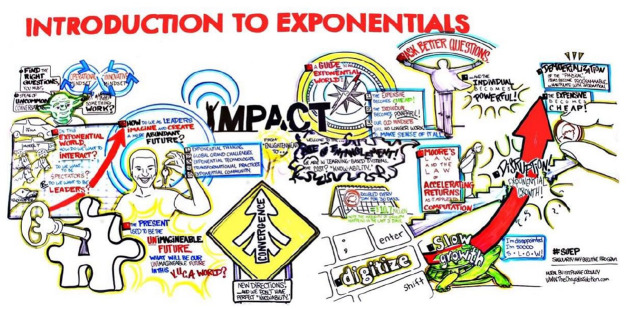
‘Introduction to exponentials’, a graphic used by Singularity University to capture transformative thinking (Constine, 2018). © Courtesy of Stephanie Papaioanu, Chrysalis Studios.

Thiel’s ‘notes on how to build the future’ illustrate how much *scaling*^1^ has become an obsession of innovation discourses and, with it, contemporary social, political and economic life at large. Perhaps most visible in the rise of platform technologies and surveillance capitalism, ‘vast scale’ has become quasi-synonymous with the success of companies that did not exist two decades ago, but now easily reach hundreds of millions of users across the globe – like Facebook, Twitter, Uber, and Airbnb. The ramifications of this scale, while hard to anticipate when these companies were in their infancy, have quickly emerged as problematic at the level of entire societies as well as globally. Frontpage news regularly zooms in on one big tech behemoth or another whose business model is challenging established socioeconomic orders, constitutional rights, political systems, and labor markets. Frequently, the strategy has been one of ‘Blitzscaling’ – a shock-and-awe tactic openly aimed at social disruption that strives to ‘achieve massive scale at incredible speed, … prioritizes speed over efficiency’, and risks ‘potentially disastrous defeat in order to maximize speed and surprise’ (Hoffman and Yeh, 2018). The current business literature and consulting industry is rife with manuals on how ‘how to create an exponential mindset’ (Bonchek, 2016) how to hack growth (Ellis and Brown 2017), or ‘How to move fast: Corporate innovation at speed and scale’ (McKinsey, 2019). Singularity University, the famous Silicon Valley breeding ground for would-be entrepreneurs, openly embraces the ambition to ‘improve the life of a billion people’ (Singularity University, 2020, see Figure 1). Social entrepreneurs are warned that ‘if you don’t know how to scale, don’t innovate’ (Seelos and Mair, 2017).

**Figure 2.**
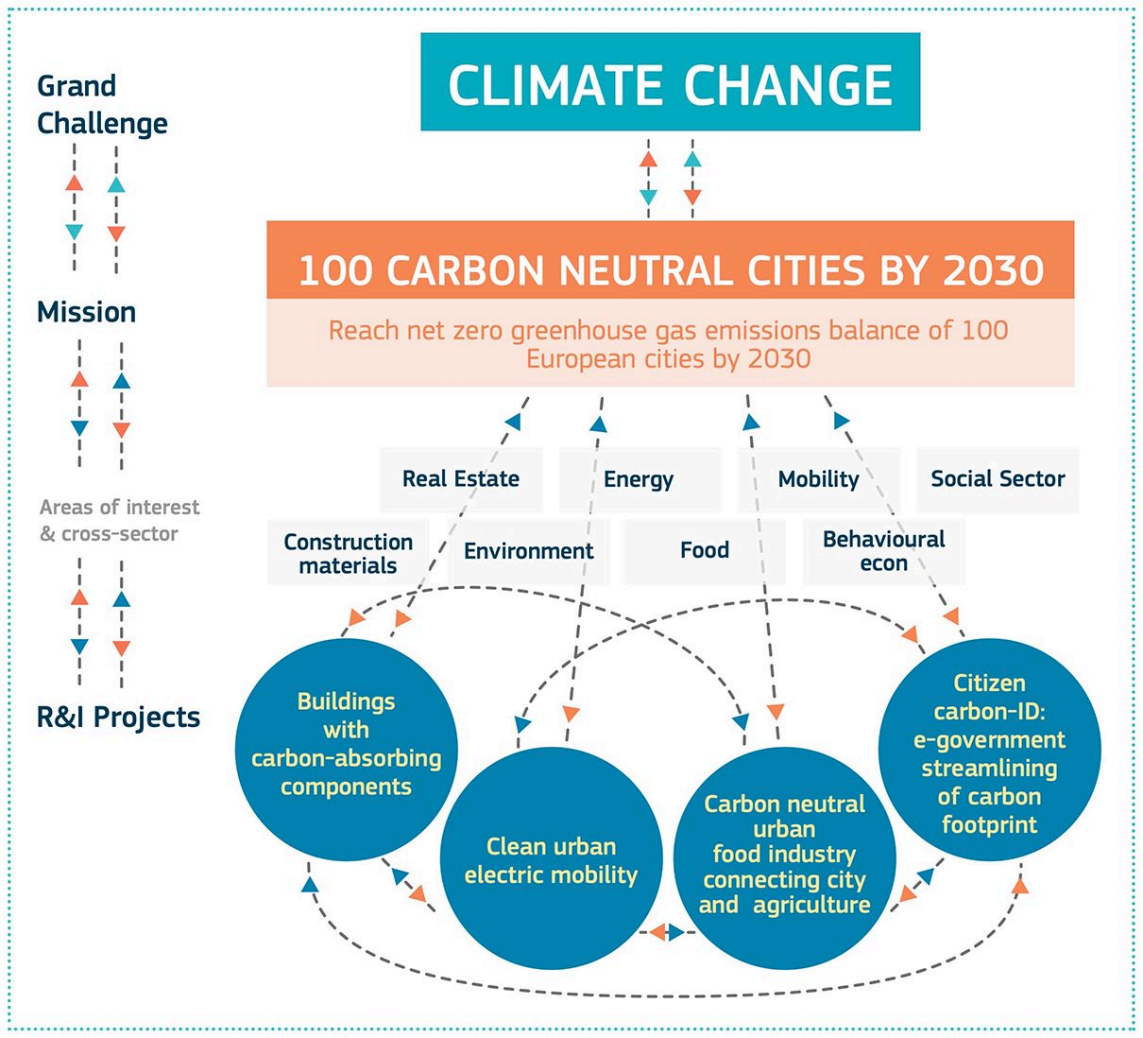
A graphic used by the European Commission to explain the logic of ‘mission-oriented innovation’. Promoted by economist Mazzucato (2018), among others, ‘missions’ serve as a meso-level organizing principle to coordinate actors and projects and direct their innovation activity toward grand societal challenges. Mission-oriented innovation has been embraced by the upcoming funding program by the European Commission, ‘Horizon Europe’.

On the public policy side, too, scalability has moved to the center stage, in keeping with a growing obsession with innovation and partly mirroring its logics (Pfotenhauer et al., 2019). Policy initiatives and public research programs are increasingly rationalized in the name of ‘grand societal challenges’, as in the forthcoming *Horizon Europe* funding program by the European Commission or in the many initiatives surrounding the UN Sustainable Development Goals. These challenges demand scalable solutions, for example in the form of ‘mission-driven innovation’ that portions socio-technical transformations into manageable and scalable pieces (Mazzucato, 2018, see Figure 2) or in corollary debates about ‘deep transitions’ (Schot and Kanger, 2018). Even ‘social innovation’ debates, usually concerned more with bottom-up dynamics and local alternatives than with megalomaniac technocratic interventions, are increasingly pushing for up-scaling (Gabriel, 2014; Musa and Rodin, 2016; Westley et al., 2014).

Scalability ambitions have, of course, always been essential to the modernist project. As Tsing (2012) writes, ‘projects that could expand through scalability were the poster children of modernization and development’ (p. 523). Tsing’s call for ‘non-scalability’ to account for ‘non-scalable diversity’ echoes a long tradition in science and technology studies (STS) of exploring the conflicts that ensue when knowledge and solutions are framed as universal rather than local and situated (Bowker and Star, 2000; Haraway, 1988; Jasanoff, 2012; Tsing, 2005). These works have studied how the modernist’s totalizing gaze tends to beget a politics of exclusion of alternative ways of living and knowing, as evidenced by decades of development policy (Ezrahi, 1990; Latour, 1993; Miller, 2004; Scott, 1999; Visvanathan, 1997a).

Yet, the current entrepreneurial appetite for quantity, speed and scale seems to have a constitutive quality of its own. While in older markets companies might grow if their products and services are successful, in the era of big tech, the aim is frequently to scale up first and profit later. Silicon Valley financiers explicitly select new ventures for their (blitz-) scalability in all-or-nothing domination strategies, which can leave the public and policymakers reeling in their attempts to account for technology’s winners and losers. For some of these technologies, the consequences of their scale is outright ‘unfathomable’, as Gillespie (2018) notes. Policy initiatives, whether in development economics or sustainability transitions, increasingly bet on experimental ‘lab’ approaches with a scalable impact logic, aimed at re-shaping some of the fundamental ways in which societies function. In this sense, ‘scale’ ceases to be an analytic category that refers to geographic reach, nested units of analysis or the marginal costs of production. Rather, as an actors’ category, it signifies an imperative and framing device for businesses, governments and NGOs alike that prescribes what seems worth doing, what the rules of engagement are and how we define problems or solutions.

In this paper, we interrogate the scalability zeitgeist as a key ingredient of contemporary political and economic ordering. Following a discussion of theorizing efforts on scaling from STS and elsewhere, we analyze three sites where scaling has become a dominant logic – platform technologies, ‘living labs’ and experimental development economics. From these diverse sites, we identify several elements of how the politics of scaling manifest in practice. We argue that the prioritization of rapid up-scaling entails a series of normative assumptions about how society and social change function (and ought to function), what kind of solutions are feasible and desirable, and who authorizes social change. We further suggest that STS’s engaged program needs to come to terms with the ‘politics of scaling’ as a powerful corollary of the ‘politics of technology’.

## What critique of scalability?

Questions of scale have been a staple of economics and management research for a long time (Arrow, 1979; Colombo and Grilli, 2010; Davidsson, 1991; Morroni, 2009; Scherer, 1980), and both economic historians and historians of technology have interrogated the social transformations, corporate models and forms of capitalisms economies of scale beget (Chandler, 1994; Hounshell, 1985; McCraw and Chandler, 1991; Scranton, 1999). Such economies of scale are linked to the rise of mass production, ‘big business’ and Wall Street, and corollary notions of ‘corporate America’ and ‘too big to fail’. One can read economists’ dogmatic rebuttal of monopoly power and its corrosive effects on market dynamics as indirect critiques of the consequences of economies of scale. This includes the domain of innovation – for example with regard to perennial questions of whether patents stimulate or stifle innovation (Heller and Eisenberg, 1998; Viscusi et al., 2000), how incumbency relates to innovation pressures (Hart, 2001) or how to reckon with antitrust in the new era of big tech (Khan, 2016; Khan and Vaheesan, 2017; Manne and Wright, 2011; Wu, 2018). Questions of scale have also been prominent in economic sociology studying the diffusion of innovation (Rogers, 2003).

According to Tsing, economies of scale can be best understood through their mechanistic essence that turns scaling into a material routine. Tsing (2012) points to ‘the ability of a firm to expand without changing the nature of what it does’ (p. 508) and to a mode of reasoning epitomized by the early colonial plantations, whose formula for smooth expansion across different landscapes was later adopted by factories during industrialization. For Tsing, certain features of sugarcane plantations have become defining features of scalability, namely alienation, interchangeability and expansion. When applied successfully, these can generate remarkable profits, as well as large human and environmental costs and ruins. It is against these ruins, or where the logic of scalability fails, that the non-scalable emerge and are made visible. Non-scalable projects, Tsing emphasizes, deserve attention, not because they are necessarily better than the scalable ones, but because they embody the kinds of transformative and mutualistic relations that scalability erases.

Tsing’s account echoes earlier work in STS, particularly in the actor-network theory (ANT) tradition, that has addressed questions of scale in science and technology by studying the enlargement of material-semiotic networks through processes of translation and attachment. Interrogating the transformative power of science, Latour (1999) famously argues that technologies effectively never leave the lab, but rather that society is gradually transformed to resemble the conditions of the lab. In this view, the lab is expanded outward to enable an up-scaling of a technology while at the same time reconfiguring society through new power structures tied to central nodes in the network – often those who control the technology. Innovation, in this sense, is a colonizing act of network expansion that depends, among other things, on the mobilization of strong allies and spokespersons (Akrich et al., 2002a, 2002b). But Tsing’s work also resonates with STS scholarship that has treated ‘non-scalability’ as a kind of foundational premise: Partly as a response to generalizing tendencies of systems theories of society and the economy, STS research has consistently emphasized the political contestation, historical contingency and interpretive flexibility that a more situated perspective engenders.

Early work in STS has been concerned with a rebuke of universalisms – whether in scientific claims to truth and objectivity (Fleck, 1981; Jasanoff, 1987; Latour and Woolgar, 1986), the transfer and expansion of technological systems (Bijker et al., 1987; Jasanoff, 1995; Star, 1999; Visvanathan, 2005), or the governance structures that accompany them (Hilgartner, 2000; Jasanoff, 1994; Wynne, 1992). The term ‘situatedness’ has become a shorthand for an analytic perspective that explicitly accounts for differences in social, cultural, political, economic, and institutional positionality (Butler, 1990; Haraway, 1988; Jasanoff, 2004). This has opened up spaces for a normative critique of hegemonic power structures and colonial tendencies that threaten to erase epistemic and political diversity (Harding, 2006; Prasad, 2014; TallBear, 2013; Visvanathan, 1997b), or ‘meaningful diversity’ in Tsing (2005: 38) language. These works relate to broader critiques of modernity premised on legibility, standardization, industrialization or massive development interventions that tend to ignore local diversity and inevitably run into resistance (Callon et al., 2009; Foucault, 1980; Scott, 1999; Visvanathan, 1997b).^2^

As STS reminds us, however, one should not think of scalability merely in terms of homogenizing acts of translation and the flattening-out of difference. For one, ‘economies of scale’ and ‘diffusion’ are specific modes of calculative reasoning for scaling up technical and economic projects that, like any other market device, depend on distributed material agency and diverse actors (Callon et al., 2007). This prompts us to scrutinize the intricate relations and persistent power inequalities that hold such hybrid assemblages together across place and time (Haraway, 2016; Ong and Collier, 2004; Strathern, 2020). For example, companies such as General Motors, Walmart or McDonald’s associate pockets of cheap labor with places of consumption in ways that make diversity an ingredient for bigness (Tsing, 2009).

For another, the success of certain scalability logics also depends on their compatibility with sticky local ways of reasoning and meaning-making that are often highly institutionalized. What seems readily scalable for scientific, technical or economic reasons might thus vary significantly across regional, cultural, and jurisdictional boundaries (Felt, 2015; Jasanoff and Kim, 2009; Levidow et al., 2007, 2013; Pfotenhauer and Jasanoff, 2017a; Winickoff and Mondou, 2017). In this sense, the expectation that things can be scaled up hinges upon the problematic assumption that technological and knowledge orders could co-evolve in a sufficiently synchronized and harmonious way with (other) existing elements of collective meaning-making, including (self-)imagination, political culture, and institutionalized (state) apparatuses of knowing, ascertaining, and demonstrating. Ensuing controversies associated with the scaling of novel technologies can thus be interpreted as expressions of these co-productionist alignments being destabilized, at least for parts of society (Hurlbut, 2015; Jasanoff, 2004, 2005).

This co-productionist perspective also foregrounds the important link between innovation, scale and imagination. Ambitions to scale up technologies and associated economic structures are linked to the creation of widely shared collective visions about the future, which makes these visions a key battleground. Hilgartner’s (2015) work on ‘vanguard visions’ explores how ‘relatively small collectives [regularly] formulate and act intentionally to realize particular sociotechnical visions of the future that have yet to be accepted by wider collectives, such as the nation. … How, for example, do “unimaginable” technological revolutions become not only imaginable but, at least for a time, plausible?’ Reversing the direction of scalability from big to small, Jasanoff (2010) analyzes the mismatch between the scientific construct of ‘global climate change’ and the capacity to imagine its meaning vis-à-vis local experience and identities. A similar observation has recently been made by Latour (2018) when talking about illusory seamlessness between the local and the global when zooming in and out in Google Earth.

These extant theoretical perspectives are useful for us to probe the current scalability zeitgeist. They invite us to ask programmatic questions such as: Which current forms, instrumental or otherwise, do scalability ambitions take in debates around innovation, entrepreneurship and ‘Big Tech’? Who are the new prophets of scalability and how do they attain credibility and wider audiences? How do present scalability logics relate to earlier modes of colonization, globalization, and ostensible homogenization? How are they able to credibly connect to the diversity of social concerns they claim to address? What is left out of scaling up processes, and to whose benefit or detriment? Where does stability in scaling processes derive from and for whom does (in)stability matter? How does the promise of scalability relate to the promise of innovation, and how do both shape present political economies? In short, how do the politics of scaling manifest and how do they relate to well-known concerns about politics of technology?

## Three scalability snapshots

The three empirical snapshots presented below derive from larger research projects in which the authors of this paper are engaged, including on the future of mobility, the mainstreaming of co-creative innovation practices and real-world experimentation. They represent a synthesizing effort of diverse empirical material that we have partly published, or are planning to publish, elsewhere. The three snapshots were specifically selected on the basis of their partly overlapping timelines and relevance to the larger conceptual questions of this paper. Although all three are not without historical antecedents, they epitomize certain elements of the politics of scaling that are particularly palpable today. For example, platform technologies have impacts that can rise to the level of entire democracies or markets within few years, making macro-political phenomena like echo-chambers, election meddling, real estate speculation, surveillance, or even genocide an explicit part of the conversation. ‘Test beds’ and ‘living labs’ promise to deliver scalable solutions in ways where possible transformations are tested before rolling them out more widely, as currently pursued in many government initiatives in energy, mobility or information technology. Experimental development economics reflect a commitment to ‘evidence-based policy’ rooted in data-driven approaches to politics that treats policy as a scale-up from local interventions. Together, the three snapshots help us identify how current initiatives envision scalable expansion and growth.

### Scalable platform technologies and the uberization of everything

Uber, one of the most notorious examples of platform capitalism and its accompanying ‘gig economy’, provides a telling starting point for an illustration of what the current scalability zeitgeist entails. The company has pursued an aggressive ‘scale first; ask questions later’ strategy, running years and billions of losses, engaging in predatory pricing (Duprey, 2020) and counting on network effects to bring exponential growth and eventual winner-takes-all profits. Within little more than a decade, the company has infiltrated the transport systems of 900 cities in 85 countries and become one of the world’s largest transportation companies, with 75 million active users and 3 million registered drivers. More recently, Uber has also pushed its platform as a solution in adjacent sectors, including food and goods delivery.

Countless upstarts aspire to be the ‘Uber for X’, aiming to build a platform that allows for scaling at negligible marginal cost and the disruption of markets while externalizing responsibilities. The story told by Uber, Airbnb and others is of a ‘sharing’ economy, with spare resources voluntarily shared among empowered owners and users for a bit of extra cash on the side. A 2017 article co-authored by Jonathan Hall, Uber’s head of policy research, and Princeton’s Alan Krueger, former head of Obama’s Council of Economic Advisors, primarily emphasizes the benefits of flexibility for drivers (Hall and Krueger, 2018). But the ‘uberization’ of the economy has led to wide controversy and occasionally fierce resistance for its starkly unequal effects. Instead of empowerment, Uber has been criticized for creating a new class of precarious self-employment outside traditional regulatory or union protection (Uber calls its drivers ‘partners’ or ‘contractors’ rather than ‘employees’). It has been blamed for a sudden displacement of taxi drivers, new patterns of exploitation (e.g. dependence through rating systems), anticompetitive behavior, and a range of data privacy issues. As with other platform technologies, the speed of scaling has meant that both the company and the jurisdictions in which it operates have been unable to attend to the myriad issues created (Gillespie, 2010).

As a result, Uber and its peers operate largely in what is generally considered a legal gray area. Uber has repeatedly been accused of sidestepping health and safety regulation. The company continues to argue that it is a technology company, not a mobility company (Conger and Scheiber, 2019), to avoid concerns about drivers and safety. This ambiguity is symptomatic for platforms that challenge conventional ways of organizing markets and governance: They claim to be technology intermediaries linking supply and demand within existing markets while actually undercutting market dynamics through a vast influx of venture capital (Kenney and Zysman, 2019) – prompting one analyst to label Uber a ‘firm-market hybrid’ (Sundararajan, 2014, 2016). Cities looking to advance their public transport systems are now recognizing that this model looks more like a problem than a complementary private sector solution. One large analysis found that Uber and its copycats have contributed to worsening congestion, decreased public transport use and negligible change in car ownership (Diao et al., 2021).

Like other platform companies, Uber’s blitzscaling efforts are closely linked to a strategy of experimentation. The company touts its ‘Experimentation Platform’, XP, and argues that ‘experimentation is a critical stage of the product lifecycle; it is the process of discovering and determining whether or not new features are successful. Given Uber’s hypergrowth, the goal of our XP is to ensure that new features roll out successfully and then return actionable analysis’ (Feng and Zhenyu, 2017). Constant variation and experimentation mean that there is no *one* Uber, just as there is no *one* Facebook. Drivers and customers will experience different versions of Uber depending on their location, usage history and socioeconomic profile. While many of these experiments concern features such as pricing, routing, display feedback or engagement that are invisible to both drivers and customers, some are also physical experiments, including the controversial use of video recording in Uber vehicles (Conger, 2019a). What is more, participants are typically unaware that they are being experimented upon. Similar to other platform technology companies, consent clauses for experimentation are usually buried deep within the terms of use, which can only be accepted or rejected wholesale. Platform experimentation has increasingly sparked controversies, for example when Facebook published the results of a psychological experiment it had clandestinely run on 689,003 users to explore how positive or negative posts in their newsfeed influenced users’ own postings (Constine, 2014).

### Scaling up from living labs and test beds for transformative technologies

In March 2020, Uber announced that it would resume public testing of autonomous vehicles (AV) in its hometown, San Francisco. Uber chose a notably humble tone in alerting the public of its plan, though not without hinting at its larger ambitions: ‘Our testing area will be limited in scope to start, but we look forward to scaling up our efforts in the months ahead and learning from the difficult but informative road conditions that the Bay Area has to offer’ (Hawkins, 2020). Uber’s plan came after a series of mis-steps, including a fatal crash in March 2018 in Tempe, Arizona, where a self-driving Uber vehicle killed a pedestrian walking her bicycle, the company’s 2017 refusal to mark its AVs as ‘test vehicles’ and the earlier loss of its license to operate in San Francisco (McCormick, 2016). The relaunch of public testing was also as a nod to investors after the company’s disappointing IPO of ‘only’ $69bn (Isaac et al., 2019), which had underscored that the deployment of AVs, with the cost-cutting potential of extracting drivers, was vital for the continuation of its business model (Conger, 2019b). Yet, later in 2020, Uber offloaded its AV division to a competitor, revealing the difficulties replacing human drivers and their attachments to the world (Tennant and Stilgoe, 2021) .

Uber’s public testing of a risky technology is representative of a broad wave of ‘living labs’ and ‘test beds’ currently sweeping across the ‘innovation’ world. In a nutshell, ‘living labs’ or ‘test beds’ are designated experimental spaces where potentially transformative technologies can be tested and developed in public under supposed real-world conditions (Engels et al., 2019; Evans and Karvonen, 2014; Gross, 2016; Kareborn and Stahlbrost, 2009; Renn, 2018). They represent a more overt, and possibly more extreme, form of many tendencies previously discussed by scholarship on the experimental nature of technology introduction (Beck, 1992; Krohn and Weyer, 1994; Lemov, 2006) and the world-making ambitions behind scientific and technological tests (Gooding et al., 1989; Latour, 1999; Laurent, 2016; Pinch, 1993; Shapin and Schaffer, 1985). Beyond mobility, living labs have been placed in domains as diverse smart cities (e.g. in Toronto’s Sidewalk Labs, Korea’s SongDo and India’s 100 Smart Cities), security (e.g. facial recognition tests in China and Germany), digital finance (e.g. Singapore’s FinTech sandbox), sustainability transitions (e.g. Masdar City in Abu Dhabi) or a mixture thereof.

**Figure 3.**
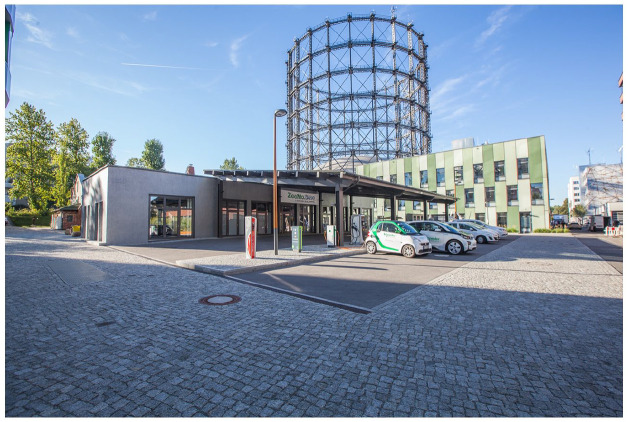
View of the European Energy Forum (EUREF) campus in Berlin, a ‘living lab’ for energy transitions and new digital and mobility technologies aimed at broader urban (and global) transformations. Technology tests include, for example, the parameters of different charging stations for electric mobility, seen in the center of the image. Courtesy of EUREF AG.

Living labs and test beds are frequently the spearheads of policy initiatives aimed at large-scale sociotechnical transitions, often in the form of public-private hybrids. For example, in 2008, the European Energy Forum (EUREF) living lab campus was launched in Berlin as a ‘an urban neighborhood model for a climate-neutral, resource-saving, smart city of tomorrow’ and ‘model for the *Energiewende* in Germany’, referring to Germany’s national flagship initiative for a sustainable energy transition (EUREF, 2018b, translation by the authors). Combining an array of energy supply, smart grid, mobility and building technologies (Figure 3), Berlin’s mayor Michael Muller heralded EUREF ‘as a space for experimentation [where] solutions are devised and tested that will touch all our lives tomorrow and into the future, [and] a place of innovation, where more than 150 companies, both established and young start-ups, exchange ideas with research institutions on important issues about the future’ (EUREF, 2018a). For many EUREF actors, testing on campus was seen as a stand-in for testing in Berlin (and other metropolitan regions in Europe), with technologies being explicitly developed ‘in a very small, scalable way’, as one engineer noted (Engels et al., 2019). Built on fenced-off private grounds and accessible only through guarded gates, EUREF was able to act with considerable organizational and regulatory leeway and partly outside Germany’s strict regulatory environment (e.g. customized charging stations or road traffic regulation). However, because of this sharp demarcation, the campus’ promise of seamless integration with its Berlin environment and stand-in character fell arguably short, prompting some citizens to call it a ‘UFO’ (Engels, 2020). As Engels et al. (2019) have argued, EUREF displays a fundamental tension between living labs conceived on the one hand as highly controlled, quasi-scientific test environments and, on the other, dynamic places representative of real-world messiness and full of unexpected ‘co-creative’ opportunities for innovation and learning.

The value proposition of living labs is closely tied to the dual promise of scalability and competitiveness. Living labs are heralded as launchpads for broader systemic change that can address ‘grand challenges’. If successful, the insights and socio-technical configurations are envisioned to provide a blueprint for wider societal roll-out and transformation. This promise has sparked renewed discussions about social testing at a meso-scale and the role of experimental attachments, and whether one could think of them also as a lever for developing scalable governance models for emerging technologies (Engels et al., 2019; Laurent and Tironi, 2015; Marres, 2020; Tennant and Stilgoe, 2021). At the same time, living labs have become a prominent avenue for cities and regions to position themselves competitively as innovation leaders and to attract tech firms (Laurent et al., 2021; Pfotenhauer, 2017). Conversely, companies are vying for municipalities as potential clients with deep pockets and transferable implementation templates. The EUREF campus, for example, has emerged as a featured stop for many national and international delegations to display Germany’s technology prowess and underscore its export potential in energy and IT. At the same time, the pressure to display market-ready solutions can force early closure of experimental technology developments in favor of quick scalable solutions. In the case of EUREF, this has led to repeated conflicts between business and engineering perspectives on how to best utilize the living lab (Engels et al., 2017).

Living lab initiatives have attracted considerable criticism, echoing in part well-known critiques of scientism from STS and beyond. Some of the main lines of critique concern the surrender of public space to commercial interests; the creation of material lock-ins through the weight of flagship demonstration projects; the fabrication of new inequalities and potential threats to democracy under the guise of openness and inclusion; a lack of attention to problems of risk governance and consent for emerging technologies; unclear ethical ramifications on large-scale public experimentation; and misguided promises of ready-made transferability and scalability. Many of these issues also apply to our next snapshot.

### Scalable policy interventions: Experimental economics and evidenced-based policy in development contexts

On October 14th, 2019, the Nobel prize in economics was awarded to Abhijit Banerjee, Esther Duflo and Michael Kremer ‘for their experimental approach to alleviating global poverty’ (Nobel Prize Committee, 2019). Pioneers in the field of experimental economics and its application to development policy, Banerjee, Duflo and Kremer spearheaded a wave of applied economic scholarship based on randomized controlled trials (RCTs) as the basis for policy intervention. This method, inspired by the natural sciences (and particularly the medical sciences), compares the differential response between a ‘treatment group’ (i.e. a population/site subjected to an intervention) and a ‘control group’ (i.e. one without such intervention) to prove or disprove the efficacy of a treatment. Following this scientific logic, effective treatments – for example, public health or training programs, a medication or vaccination fix, or cheap sanitation technology – are then meant to scale up to achieve global impact at the level of grand societal challenges (Tollefson, 2015). In the words of MIT’s Abdul Latif Jameel Poverty Action Lab (J-PAL, 2020b), an institution that is the brainchild of Banerjee and Duflo, ‘over 400 million people have been reached by programs that were scaled up after being evaluated by J-PAL affiliated researchers’.

By grounding development policy in scalable local experiments, the three Nobel winners and the institutions they inspired are part of a broader wave of development scholarship that seem to upend decades of received wisdom on development policy. Instead of understanding development as a macroeconomic domain driven by national indicators in which international funders need to come to terms with local political and institutional conditions, they argue that development interventions can be purely ‘evidence-based’ and free from (or at least agnostic to) such political considerations thanks to the scientific authority of experimentation: ‘What works’ clearly speaks for itself. Once evidence of efficacy has been established, the intervention can then be harnessed beyond the experimental site to trigger wider policy initiatives (Bold et al., 2013). The RCT approach mirrors, in a bottom-up fashion, long-standing top-down tendencies by international development organizations such as the World Bank who, according to Tsing (2012), tend to envision scalable activities that ‘could be spread to other villages without changing project elements’. It does so by reducing the initial ‘project’ to a limited set of parameters fit for experimentation and expanding the envisioned impact of the would-be tested solution to nothing else than ‘solving poverty’ (Stein, 2019).

One of the regularly cited examples of projects run by J-PAL is an experiment conducted in the Delhi area that demonstrated that deworming young children was beneficial to school participation, thus inspiring deworming policies in Africa and India (Abdelghafour, 2017). This experiment alone has solicited substantial debate about the nature of data generated by RCTs, and particularly efficacy and scalability claims associated with it (see Evans, 2015). Critics of RCT use in development policy have further pointed to their inherently contradictory premise that that their findings are context-specific yet scalable (Bédécarrats et al., 2019, 2020; Deaton and Cartwright, 2018), thus casting doubt on the key premise of initiatives like J-PAL (2020a) ‘to reduce poverty by ensuring that policy is informed by scientific evidence’.

Proponents of experimental economics tend to counter such criticism with a claim of efficiency: The evidence created by RCTs allows policy-makers to put scarce development resources to optimal use. This situates experimental economics in a wider trend of evidence-based policy-making that has also tapped into behavioral economics. For example, in the UK, Nesta runs as Innovation Growth Lab that promotes ‘a more experimental approach to innovation and growth policy’ by running and sponsoring RCTs (IGL, 2020). The Behavioural Insights Team started as small unit in the UK government and grew into ‘a global social purpose company’ with the mission to ‘generate and apply behavioural insights to inform policy, improve public services and deliver results for citizens and society’ (BIT, 2020). The logic is remarkably consistent: An initial experimental intervention is intended to produce outcomes that can generate scaled-up policy change. The Behavioural Insights Team borrows venture capital language in its claim to create ‘policy unicorns’, that is, massively scalable behavioral nudges that ‘had an impact of more than £1 billion’ (Halpern, 2019).

Soon after Banerjee, Duflo and Kremer received the Nobel Prize, Sanjay Reddy, a professor of economics at the New School argued in *Foreign Policy* that even though RCTs had become dominant in development economics, they were often conducted ‘at the expense of the world’s poor’ (Reddy, 2019). At a time of nearly unanimous celebration, Reddy and others exposed some of the issues of experimental economics in development contexts, such as the ethical implications of involving human subjects in experiments^3^ (Rayzberg, 2019), the scientific robustness of the ‘evidence’ produced (Bédécarrats et al., 2019), and the unintended consequences of scaling up isolated initiatives without taking side effects into account (Abdelghafour, 2017; Jatteau, 2013). These discussions invite us to explore the radical re-definition of development policies that RCTs imply. They also suggest that reasoning in the terms of local initiatives meant to be scaled up has profound consequences in the world of development.

## Exploring elements of the politics of scaling

As illustrated by our three snapshots, scalability is currently featured prominently in initiatives of innovation, entrepreneurship and public policy – cutting across regions, sectors, and scientific domains. While there are many ways to analyze these cases, three analytic themes seem particularly salient to the politics of scaling and its effects: Their shared emphasis on tech solutionism, public experimentation, and future-oriented valuation practices.

### Solutionism

A first element shared by our three snapshots is their affinity for solutionism – that is, the framing of problem diagnoses in response to readily available, often technological solutions. Solutionist thinking is closely related to notions of ‘technological fixes’ (Johnston, 2018; Rosner, 2004; Volti, 2006) and ‘technology optimism’ (Tiles and Oberdiek, 1995), which cast a long and consequential shadow over the history of engineering and public policy, and which have gained renewed currency in the area of tech entrepreneurship. To authorize certain problem diagnosis, solutionism tends to draw on technical languages, visionary promises, and other forms of exclusive expertise to assert causal relationships and credibility. Problems are cut into smaller, discrete pieces that warrant ready-made solutions, which are in turn owned or controlled by specialized organizations or individuals (entrepreneurs, innovators, scientists, innovators etc.). Alternative problem framings that might benefit different groups of actors are thus frequently sidelined. Morozov (2013) analyzes how tech companies, and specifically Silicon Valley-based unicorns, craft misleadingly narrow problem diagnoses around available gadgets. Different solutions to this problem are then often presented as equivalent to one another, when in their socio-economic reality they are clearly not (e.g. addressing obesity as a public health concern by either regulating the food industry or by using a self-tracking nutrition app). Similarly, Pfotenhauer et al. (2019) explore a ‘deficit model’ in innovation policy where a lack of innovative solutions is regularly diagnosed as the reason for why we cannot address societal challenges, thus treating innovation as a kind of panacea across social and policy domains (see also Pfotenhauer and Jasanoff, 2017b).

Platform technologies, living labs and experimental economics all frame their quest for scalability in the language of solutionism. Here, the commitment to a scalable solution affects which problems are deemed relevant – that is, those that can be addressed through a scalable solution – and the type of intervention that is expected to follow. Platform technologies such as Uber propose to solve a large-scale mismatch between local supply and demand for individual transportation through a decentralized, scalable matching platform. Yet, the controversies surrounding Uber reveal the multiplicity of possible specific problem definitions (e.g. inefficient matching of potential drivers and potential clients, insufficient public transportation infrastructures, underutilized private cars, corrupt taxi markets, excessive labor market regulation) and corollary framings as to what kind of solution the company is actually providing (a software, a mobility service, a platform for independent contractors, a taxi company with thousands of employees, a complement to public transportation, a brazen attempt to undercut minimum wages, and diverse regulatory standards etc.). Here, the company’s own framing of the problem and the solution, as well as the suppression of alternative framings in public debates such as the California Prop 22 decision (Uber Newsroom, 2021), can be seen as a purposeful (mis)direction in favor of scalability and away from less scalable solutions. Some of Uber’s investors would argue that the real problem the company is tackling is the expectation of investors for exponential growth, inhibited by the inconvenience of social-democratic laws, minimum wages, employee benefits and organized unions.

While Uber was pitched as the solution for transport mismatches, EUREF was envisioned as a stand-in for urban energy transitions across Berlin and many other urban areas in Germany and Europe. It took on grand, messy societal challenges like sustainable energy provision or climate change and carefully constructed its activities as plug-in solutions with scalability potential, including micro-smart grids, e-scooters, lamp post charging stations, and charging location apps that can help overcome fears of a shortage of charging stations for potential customers of electric vehicles (Engels et al., 2017). As Wentland (2018) has shown, over the course of 10 years EUREF repeatedly re-framed its vision in response to shifting policy trends and problem diagnoses, including the *Energiewende*, Smart Cities or a Green New Deal, as well as broader imaginaries of an automobile nation, sustainability or energy security. Across these shifting societal challenges and problems, the living lab remained a demonstration site for a similar set of technologies portrayed as potential solutions and a mechanism meant to assure wider public acceptance. Engels et al. (2019) have argued that EUREF and other living labs serve as instruments to test and stabilize future societies around a predefined set of technological premises based on rather specific visions of the future, which raises questions about the inclusiveness of such visions.

With its grand aim to ‘solve poverty’, experimental economics, too, treats societal challenges as discrete, isolated problems that warrant localized interventions. In particular, many interventions focus on cheap technological fixes (such as the distribution of de-worming pills or low-cost water filtration) to achieve what decades’ worth of development policy could not. In this sense, the solution to the malaise of poverty-ridden regions of the Global South is higher skills levels of the population to allow them to participate in the global knowledge economy, made possible by increased school attendance, in turn made possible by decreased worm infections, guaranteed by drugs manufactured in another country. While all of these improvements are important achievements, they reduce the problem of a continent’s under-development (a framing that is in itself highly problematic) to something that can be tackled through a pill, sidestepping more complicated systemic challenges such as a consistent underinvestment in teachers and school buildings, dysfunctional governments or lack of legal protection against child labor, let alone the configuration of global value chains, trade agreements, or migration policies.

### Experimentalism

Second, the promise of scalability hinges on the assumption that the efficacy of scalable solutions to ‘grand societal challenges’ can be established locally and, once proven effective, rolled out society-wide – addressing essentially the same problem through more of the same. The desired proof is in many cases closely tied to the notion of scientific experimentation and its appeal of objectivity – which are especially visible in living labs and RCTs. Our scalability snapshots fit well with a broader interest in laboratory practices and experimental knowledge beyond traditional laboratory settings (Engels et al., 2019; Laurent, 2017; Millo and Lezaun, 2006; Papageorgiou, 2017; Stilgoe, 2016).

Like all scientific experiments and tests (Latour, 1988; Pinch, 1993; Shapin and Schaffer, 1985), our three scalability cases can be read as (staged) public demonstrations – including toward companies, investors, regulators, future users or the citizenry at large – who are enrolled to attest to the credibility of the performance. Experimental sites for scalability may be created from scratch (e.g. automotive test tracks with artificial obstacles) or retrofitted onto a building, a campus, a village, a city, or a landscape (Guridi et al., 2020; Laurent et al., 2021; Laurent and Lechevalier, 2019). Models for how to turn places into experimental sites are increasingly circulated, similar to how innovation and policy practices travel around the globe (Pfotenhauer and Jasanoff, 2017a; Voß and Simons, 2014, 2018). Some of Uber’s experiments have taken place invisibly, powered by its software and with its users as subjects, and some have treated the world as a physical lab, as with its high-stakes prototyping on public roads in Arizona, with bystanders as unwitting participants (Stilgoe, 2020). The EUREF living lab was implemented on a privately owned, fenced-off campus within Berlin. J-PAL actively chooses sites as well as treatment and control populations for experimental RCT-type interventions.

However, these demonstrations are rife with frictions and contestations, including over questions of participation, power, foregone alternatives and the distribution of costs and benefits. Uber’s rapid diffusion through cities around the world is a signal to investors that the promised monopoly is indeed attainable. Yet publics around the globe can also witness how Uber’s experiments have overwhelmed cities and consistently squeezed margins for drivers, leaving a trail of controversy in their wake. The EUREF living lab is a highly visible panoply of demonstration efforts with considerable policy and economic traction, regularly featured as an international showcase for the superiority of German sustainable energy technologies and proof that the *Energiewende* is indeed feasible. The campus has become an obligatory passage point for many international delegations and a favored stage for national sustainability policy debates. At the same time, EUREF has seen internal controversies over how open-ended these developments really are, how quickly closure in R&D processes ought to occur, how big its transformative impact on Berlin or Germany has been, how the infrastructures needed for some demonstrations may prevent alternative projects, and how innovation districts are subject to investor logics and gentrification (Engels, 2020). J-PAL mobilizes the authority of controlled experiments to certify efficacy claims of policy intervention as scientific and hence potentially universally valid (given sufficient control over the boundary conditions). J-PAL’s researchers ‘help partners scale up effective programs’ to entire regions and nations (J-PAL, 2020c). But J-PAL, too, has been unable to avoid controversies about the generalizability of its findings to other regions with different conditions and the ethics of large-scale social experimentation.

Our three snapshots also point to ways in which experiments create path-dependencies, giving preference to some solutions over others while explicitly aiming for their scalability. From this perspective, the objective of the experiment is connected less to the possibility of replication of test results, or the relative efficacy of different interventions, than to gaining a head start for the extension of certain commercial solutions in tandem with processes of valuation and economization. Uber’s algorithmic experiments explicitly guide future development and expansion efforts with a view toward enhancing a monopoly position. Likewise, the company’s experiments with AV were a clear signal to investors. EUREF, which hosts many leading German energy companies, explicitly aims to become a model for Berlin and beyond. J-PAL’s approach is to ‘Innovate, test, scale: Replicating and expanding a successful evaluated pilot to similar contexts’ (J-PAL, 2020). This points to the possibility of the promise of extension becoming an end in itself for experimentalism, counting on ensuing path-dependencies and on making some experiments ‘permanent’ (Karvonen, 2018).

### Future-oriented value

Finally, the promise of scalability is also a promise about future value, frequently in ways that blur social, economic, and moral value propositions. Uber’s hunt for monopoly profits is at once a proposition about sharing underutilized resources and enabling beneficial side-gigs for driver. Living labs like EUREF propose to revolutionize energy systems and evade the prohibitive social and economic costs of climate change by testing new products and services *in situ* as if the former had already diffused throughout society. Public health improvements for developing countries based on RCTs reiterate the problematic promises of development aid in conjunction with raising spending efficiency for policy makers and export markets for companies. In all three cases, the proposed value is largely speculative and tied to a carefully curated set of expectations about forthcoming transformations. What is more, this expectation is often tied to a corollary set of financial mechanisms that can derive revenues from these transformations. As such, the scalability zeitgeist resonates with the rise of shareholder value logics in management theory that link social value and responsibility to profit maximization (Jensen, 2001; Lazonick and O’Sullivan, 2000). It further resonates with current trends toward assetization and technoscientific rentiership, which turn present and future control over things – land, skills, people, rights etc. – into durable revenue streams for investors (Birch, 2020; Birch and Cochrane, 2021; Birch and Muniesa, 2020), and promises of economic opportunity into tradable entities (Sunder Rajan 2006, 2017). This link between transformation, scale and revenue is well captured by Peter Diamandis, co-founder of Singularity University, who evangelizes about the need for entrepreneurs to have a ‘Massively Transformative Purpose (MTP)’ (Diamandis, 2017). In connecting financial value explicitly with solutionism, he argues: ‘The best way to become a billionaire is to solve a billion-person problem’ (Diamandis and Kotler, 2016). These connections help explain why ‘scaling’ is increasingly treated as a goal in its own right, accepting the implied disruptive quality of innovation and de-emphasizing the details of the envisioned transformation as well as who gets to decide about them.

Accordingly, Uber’s scalability ambitions and those of its peers are tied up with a venture capital logic that expects disproportionate profits from one blockbuster out of a portfolio of many start-ups. Within this logic, each company must make grand claims to scalability while knowing, along with their investors, that most will fail or end up as components of others’ systems. Uber has become a symbol of speculative investments in unison with a move-fast-and-break-things attitude in the hope of future monopolistic revenue (Kaminska, 2016). Living Labs such as EUREF position their activities as both morally urgent and financially promising. EUREF promises to unlock both a clean, sustainable urban energy future and lucrative, growing clean-tech markets for German companies and investors. The research campus itself is a stock market-listed corporation that has raised ‘200 million euros of equity capital … and twice that much as debt’ and that has tried to frame its mission with ‘economic clarity and a hands-on controlling system’ (EUREF, 2018a). This fits the analysis that the *Energiewende* was from its inception as much a project of securing German economic and technology leadership as a response to climate change (Joas et al., 2016). Development interventions, including those by J-PAL, invoke a future where poverty is ‘solved’ and millions have been successfully integrated into global markets, presuming that governments and other funders spend resources in the present. Here, experimental interventions in developing countries serve simultaneously as an exploration of potential markets for innovations such as pharmaceuticals or financial transaction apps. Non-monetary values are thus part and parcel of the legitimacy of an extension of the expectations of future values to new territories, such as those of development and social policies.

The politics of scaling are thus an intensely future-oriented business that often gets entwined with claims to common good. What is at stake in visions of transformation and scalability – and the valuation instruments and practices that undergird them – is control over the future (see also Hilgartner, 2015, 2017), as shown by the tensions and controversies that scalability cause. Uber is betting on a future where its billions of sunk venture capital have finally outcompeted all rivals to enable monopoly profits, and where the economic dependences and the promise of comfort can be mobilized to outweigh concerns about workers’ rights and exploitation – as seen in the Prop 22 ballot controversy in California. The commercial orientation of living labs has often led to internal debates about trade-offs between speculative and socially inclusive research projects on the one hand, and the coveted financial returns on technologies or the value of the campus itself on the other. Interestingly, while EUREF has arguably fallen short of the promise of transforming Berlin through a roll-out of sustainable energy, IT and mobility technologies, it has attracted considerable attention as a scalable model to stimulate innovation, entrepreneurship and regional revitalization, which other regions have now turned to emulate (Engels et al., 2019). When experimental economists test development solutions for the sake of their future extension rather than present benefits in the experimental sites, they anticipate future social and economic value – possibly for an undefined general category of ‘the poor’ as future beneficiaries who are part of the promise of global extension.

Through the lens of scalability, then, well-known questions of power and distributive justice around valuation practices thus take a new pressing form: What role does scalability play in plausibilizing expectations about socioeconomic transformation and associated revenue streams? Which technical-instrumental form do promises about scalability take, underwritten by which expertise? What are the social, political, and material pre-conditions of the world in which promises of scalable solutions – whether by tech billionaires, scientists, or policy-makers – are not just plausible, but sought after? Who needs to be convinced about the value of scaling? Who gets to shape whose needs and vulnerabilities are represented and valued in up-scaling endeavors? The subjects of scalability experiments, while regularly asked to participate in the risks of such initiatives – whether as precarious drivers in the gig economy, pedestrians in a testbed for autonomous driving or experimental subjects in developmental interventions – are rarely asked to participate in the articulation of value propositions.

## The constitutional quality of scaling: Coming to terms with the scalability zeitgeist

Our goal in this paper has been to draw attention to the widespread commitment to ‘scaling up’ across very different sites and domains. Our analysis has shown how such scaling efforts share a number of characteristics that shape our world: in how entrepreneurs and policy-makers frame societal problems and propose transformative solutions, in the configuration of public experiments that test and materialize future ways of living ‘as if’ consensus about this future had been reached, and in business plans and valuation practices where the rewards of quick fixes and rapid domination outpace those of careful design. Our cases provide illustrations of a desire for ‘blitzscaling’, which considers the speed of extension as an objective in itself, necessary to conquer markets or solve global problems. While this desire provides an explicit blueprint for companies like Uber, it serves as an implicit promise for living lab innovators and development economists – a promise that is never fulfilled, yet proves useful to convince partners, investors, or donors of the great transformational potential of local interventions. In all cases, the combination of solutionism, experimentalism, and future-oriented value not only frames collective issues and defines how to act on them, but also distributes the ability to intervene on these issues in unequal ways.

This sensibility reveals limits with the current framing of mainstream technological critique. For example, anti-trust debates about breaking up ‘Big Tech’ (Manne and Wright, 2011; Wu, 2018) or Zuboff’s (2019) analysis of surveillance capitalism tend to focus on where we are, rather than how we got here. That is, they sidestep the critical process dimension of up-scaling and hence questions about the conditions under which certain actors were able to acquire so much power so quickly. Likewise, the enthusiasm with which big tech companies have embraced ‘ethics’ approaches to ostensibly deal with the corrosive effects of their scale (e.g. in the problematic ‘AI ethics’ initiatives of Google and Facebook) suggests that this is a convenient mode of self-critique that seeks refuge in universal principles and post-hoc institutional fixes that apply independently of scale, obfuscating the fact that the questions that can be asked early on might be quite different. The STS literature has often looked for the ‘politics of technology’ in initial material configurations and ‘design ethics’, which has led to a push for deliberation ‘upstream’ in the R&D process (Wilsdon and Willis, 2004). Similar logics apply in current ‘ethics-by-design’ proposals in AI, neurotech or gene editing (IEEE, 2020; Poel and Robaey, 2017; Schwarz-Plaschg et al., 2017). In this sense, initial design considerations will inevitably affect the conditions of possibility for up-scaling and how power will get redistributed in the process. Yet the politics of technology clearly shift when expanding beyond the initial design or testing stage, and experiences made during up-scaling regularly feed back into the re-design of business models, as in the multi-layered experiments of Uber XP, the continuous data collection in AV open-road testing and machine learning, or impact assessment for J-PAL’s interventions (e.g. Stilgoe, 2018, 2019). What is more, scalability efforts inevitably straddle different social, cultural, and jurisdictional contexts, calling for a more dynamic understanding of the design ethics vis-à-vis the public good and the variegated polities of technology politics.

The politics of technology, in short, need a corollary politics of scaling. Taking the effects of scaling up seriously would require scrutiny of normative, epistemic and material configurations *before* things scale, *while* they scale, and *after* they have scaled. As well, it requires a critical appraisal of the political-economic conditions that make scaling plausible and desirable in the first place, and an analysis of how these conditions shape what is expected to scale and for whose benefits.

**Figure 4.**
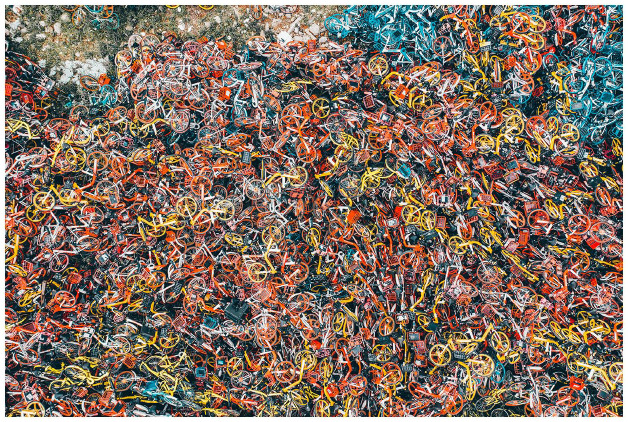
A pile of shared bikes in Shanghai. © Getty Images.

The three elements we highlight in our paper open up perspectives for future scholarship and policy interventions aimed at the politics of scaling. First, greater attention to patterns of solutionism can help us identify when, how and by whom problem spaces are configured to enable scalable solutions. Hilgartner (2017) has fruitfully interrogated nascent ‘knowledge-control regimes’ that allow vanguards to configure ‘categories of agents, spaces, objects and relationships among them’ in the name of prophetic revolutions to affect the distributive consequences of innovation. Along those lines, by giving scalability a preferential status among selection criteria for potential solutions, the usual checks and balances – for example, risk assessment, regulatory scrutiny, public consultation or protest – may be sidelined by sheer momentum or through locally justified ‘exemptions’. This puts the spotlight on moments where actors draw boundaries between supposedly ‘scalable’ and ‘non-scalable’ parts of reality. Why, for example, is the value proposition of ride-hailing companies linked to the total number of global rides, not the livelihood of healthy local communities? Why is the scaling-up of electric vehicles seen as a more plausible response to climate change than the redesign of urban spaces around or less car-centric forms of mobility? How much can de-worming pills really challenge deeply rooted global patterns of inequality and dependency? These are the kind of questions that are crucial to ask for global problems to be addressed in ways that are meaningful for the many, and not just profitable for the few (Figure 4).

Second, public experiments today almost inevitably raise questions about responsible governance. Attempts at ‘disruptive’ or ‘transformative’ innovation increasingly work within, or even purposefully create, zones of legal ambiguity and exception that may become institutionalized and permanent. Uber, Facebook, Google, and others continue to insist that existing laws, regulations, and norms on such issues as employment, privacy or freedom of speech do not apply to them because their innovative products and services operate in unprecedented legal territory. Living labs often purposefully lower regulatory burdens to experience the effects of novel technologies in real life ‘as if’ these technologies had already been proven safe and effective (Engels et al., 2019; Laurent, 2016; Laurent et al., 2021). Experiments in development economics enroll people in large-scale experiments with unclear answers to questions of consent, risk or benefit sharing. In all cases, innovators and policymakers tend to see existing regulations and norms as a hinderance to scaling. Scalability thinking thus fits in a highly problematic framing that sees innovation as the ‘driver’ of change and regulation ‘lagging’ behind (Hurlbut et al., 2020; Jasanoff, 2011; Pfotenhauer and Juhl, 2017). A critical analysis of the politics of scaling must broaden the space of reflection about how innovation and regulation can relate to each other beyond such one-dimensional framings.

This focus on the relationship between scaling and individual and collective rights also raises questions about consent. As Engels et al. (2019) note, population-scale testing in medical studies typically needs to meet very high ethical standards and requires some form of informed consent. In contrast, many current forms of public experimentation lack meaningful consent procedures, let alone clear rules for benefit sharing. Many ‘co-creative’ settings purposefully blur the differences between experimenters, test-subjects, consumers and citizens. A more optimistic reading on the proliferation of public experimentation might focus on opportunities for collective explorations of emerging issues, making experiments possibly more valuable for democratic-deliberative reasons rather than technical-instrumental ones (Callon et al., 2009; Marres and Stark, 2020; Tennant and Stilgoe, 2021). By extension, one could see experiments as opportunities to co-develop technologies, governance and social resilience in tandem, for example by treating living labs not as low-regulation zones, but as zones of greater scrutiny. Yet this would require a degree of reflexivity and political will that current experimental sites lack.

Third, and related, we should explore how the valuation mechanisms that currently incentivize scaling can be pluralized. Ours is not necessarily an argument against scale or against scaling. Nor do we argue that non-scalability is inherently good. Rather, our concern is that scaling, and in particular blitzscaling, often obscures and privatizes consideration of the opportunities and uncertainties of innovation, closing off a multitude of possible promising directions in favor of a few (Frahm et al., 2021; Leach et al., 2010). To that end, the routes of extension taken matter. Scaling up is never just ‘more of the same’. For example, letting autonomous vehicles out of a test environment and into our public road system does not just require additional hardware and additional data. It may also mean challenging existing ways of driving, policing, governing, planning, and kitting out local roads and communities – in ways that will inevitably bump up against institutionalized social norms and safeguards. The promise of extension thus hinges upon the continuous translation of heterogeneous actors, concerns, norms and things into a single scalability proposition that implicates experimental research ethics, finance, risk assessment, social justice, urban planning, anti-trust, international trade and more. A critical analysis of the politics of scaling can help us to get better at anticipating, understanding and navigating these junctures and tipping points in up-scaling processes where mechanisms that protect the most vulnerable come under pressure.

A key question for a critically engaged social science program on scalability might thus be whether we can get away from one-off engagements with the politics of technology (and the politics of policy), and instead foster a more continuous engagement with these politics across different scales – and, in the processes, figure out collectively what the scalability objective is about. We contend that such exploration is necessary lest we accept absence from some key sites where decisions about the future are presently made.

Some instruments from the STS engagement toolkit have emphasized the process dimension of research and innovation, including constructive technology assessment (Schot and Rip, 1997), anticipatory governance (Barben et al., 2008), midstream modulation (Fisher et al., 2006), or suggestions around ‘stage-gating’ in responsible research and innovation (Stilgoe et al., 2013). But their application to a politics of scaling would require differentiating better how such instruments can be applied at different scales, and, more importantly, how they can help us to problematize the injunction to scale things up. That is, can our practices of inclusion, deliberation and reflexivity work at different speeds and scales – and can they open up the range of speeds and scales that innovators, regulators, and concerned publics are engaged in? How can we rethink modes of deliberation and engagement to accompany innovations all the way through, beyond ‘design ethics’ and post-hoc regulation, in ways that never lets up pressures of democratic accountability? How can we embed our STS toolkits into settings where scalability work is currently done invisibly and with little scrutiny, especially in the private sector and start-ups (Pfotenhauer et al., 2021)? How could we, for example, scale public engagement activities with a company like Facebook from its inception in a Harvard dormitory to its 2.5 billion user quasi-utility with a data monopoly? How can we open up scalability processes to different space and time frames, allowing for debate on what should be replicated or extended, and by whom? How can we, rephrasing Collingridge’s (1980) dilemma of control, govern emerging technologies *from* the point where changes are easy but consequences are unclear, *to* the point where the consequences are mostly clear but change has become hard, in a way that always emphasizes plurality of options and configurations along the way (Winickoff and Pfotenhauer, 2018)?

In a way, the scalability zeitgeist forces us to look at the relationship between innovation and the public good more explicitly as a dynamic problem. Coming to terms with this zeitgeist will require strengthening the continuities between different modes of collective action across space and time. It will also require stressing the discontinuities that are being glossed over by proposing scalable solutions. To that end, we may take a hard look at a key lesson from the climate change debate: Why *don’t* some things scale easily? Scaling up our collective response to climate change has been notoriously difficult because people neither agree on problem definitions nor solutions; because the effects of climate change and mitigation efforts translate into different real-world experiments depending on location; and because different constituencies in the global political economy don’t agree on how to value what. Any site where scaling is made to look easy should thus raise red flags about a likely lack of comprehension or inclusiveness of perspectives.

## Notes

1. We use the terms ‘up-scaling’, ‘scaling’, and ‘scalability’ somewhat interchangeably, depending on context.

2. STS research on scale has evolved in conversation with work in other academic fields such as geography. At the interface with cultural, human and political geography, Barry’s work has addressed the controversies and geopolitics surrounding the scale of technical and knowledge infrastructures (Barry, 2001, 2013). This work taps into decades of research centrally concerned with questions of scale, particularly as part of globalization discourses and as a methodological yardstick for geographic inquiry (e.g. Herod and Wright, 2008; Sheppard and McMaster, 2008). Researchers in geography have, for example, investigated socio-material practices of association (Cox, 1997), subtle processes of socio-cultural reproduction and consumptions (Marston, 2000) and the historical-material ‘producedness’ of nature-culture that begets its own politics of scale beyond traditional Marxist readings of economic power and material determinism (Swyngedouw, 2008). They have also engaged in their fair share of controversy about the utility and ontological status of the concept of scale, for example in favor of flat ontologies or praxeological approaches (Hoefle, 2006; Marston et al., 2005).

3. This echoes well-known challenges from the history of (medical) randomized controlled trials in terms ethics, bias, and social justice concerns (Baele, 2013; Deaton and Cartwright, 2018; Petryna, 2009; Teele, 2014; Wahlberg and McGoey, 2007).
